# Urine biomarkers enable pancreatic cancer detection up to 2 years before diagnosis

**DOI:** 10.1002/ijc.34287

**Published:** 2022-09-23

**Authors:** Silvana Debernardi, Oleg Blyuss, Daria Rycyk, Kirtiman Srivastava, Christie Y. Jeon, Hui Cai, Qiuyin Cai, Xiao‐Ou Shu, Tatjana Crnogorac‐Jurcevic

**Affiliations:** ^1^ Centre for Cancer Biomarkers and Biotherapeutics, Barts Cancer Institute Queen Mary University of London London UK; ^2^ Centre for Prevention, Detection and Diagnosis, Wolfson Institute of Population Health Queen Mary University of London London UK; ^3^ Department of Medicine Cedars‐Sinai Medical Center Los Angeles California USA; ^4^ Division of Epidemiology, Department of Medicine Vanderbilt University School of Medicine Nashville Tennessee USA

**Keywords:** early detection, pancreatic cancer, prediagnostic samples, urine biomarkers

## Abstract

The poor prognosis of pancreatic ductal adenocarcinoma (PDAC) is mainly attributed to late diagnosis. We assessed the predictive performance of our previously reported urine biomarker panel for earlier detection of PDAC (LYVE1, REG1B and TFF1) in prediagnostic samples, alone and in combination with plasma CA19‐9. This nested case‐control study included 99 PDAC cases with urine samples prospectively collected up to 5 years prior to PDAC diagnosis and 198 matched controls. The samples were obtained from the Shanghai Women's Health Study (SWHS), the Shanghai Men's Health Studies (SMHS) and the Southern Community Cohort Study (SCCS). The urine biomarkers were measured by ELISA. Plasma CA19‐9 was quantified by Luminex. Multiple logistic regression and Wilcoxon rank‐sum and Mann‐Whitney test were used for analysis. The internal validation approach was applied and the validated AUC estimators are reported on. The algorithm of urinary protein panel, urine creatinine and age named PancRISK, displayed similar AUC as CA19‐9 up to 1 year before PDAC diagnosis (AUC = 0.79); however, the combination enhanced the AUCs to 0.89, and showed good discriminative ability (AUC = 0.77) up to 2 years. The combination showed sensitivity (SN) of 72% at 90% specificity (SP), and SP of 59% at 90% SN up to 1 year and 60% SN with 80% SP and 53% SP with 80% SN up to 2 years before PDAC diagnosis. Adding the clinical information on BMI value resulted in the overall improvement in performance of the PancRISK score. When combined with CA19‐9, the urinary panel reached a workable model for detecting PDAC cases up to 2 years prior to diagnosis.

## Graphical abstract

What's new?

Exceptionally high lethality of pancreatic cancer is due largely to the late stage at diagnosis. Analyses of candidate biomarkers with potential for early detection of pancreatic cancer have been inconsistent. Here, the authors examined the predictive performance of a urine biomarker panel consisting of LYVE1, REG1B and TFF1, alone and together with the existing marker CA19‐9. The urinary panel complemented plasma CA19‐9, enhancing its performance in detecting cases up to 2 years prior to cancer diagnosis. The urinary biomarker panel and affiliated risk score are promising noninvasive tools for stratification of patients at risk of pancreatic cancer.

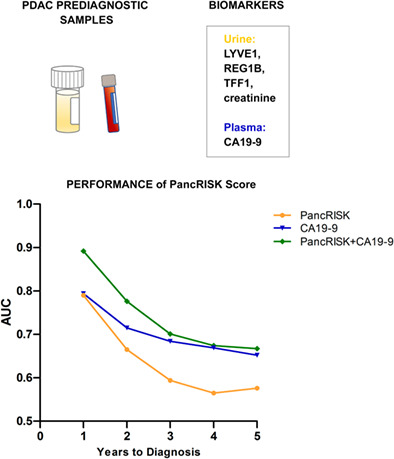

**Abbreviations**
AUC: area under the curve
BMI: body mass index
CI: confidence interval
CV: coefficient of variation
DX: diagnosis
FC: familial cancer
IQR: interquartile range
OR: odd ratio
PDAC: pancreatic ductal adenocarcinoma
ROC: receiver operating characteristic
SCCS: Southern Community Cohort Study
SMHS: Shanghai Men's Health Studies
SN: sensitivity
SP: specificity
SWHS: Shanghai Women's Health Study

## 1 INTRODUCTION

Pancreatic ductal adenocarcinoma (PDAC) is one of the most lethal cancers, with median survival of 5 to 6 months and with only 11% of individuals surviving more than 5 years in the United States.^1^ The early symptoms are nonspecific and often intermittent,^2^ hence over 80% of cases are diagnosed at advanced stages, when the tumour is already locally advanced or spread to other organs.^3^ Improving the early detection of PDAC would significantly impact patients' prognosis as a survival of >60% has been reported after incidental diagnosis of tumours when they were still confined to the pancreas and smaller than 2 cm.^4^ In addition to commonly used CA19‐9,^5^ none of the numerous candidate markers from serum/plasma^6^, ^7^, ^8^, ^9^ and less frequently urine^10^, ^11^, ^12^, ^13^ that are specific for PDAC have performed to a level for widespread screening tool. This highlights the number of challenges in the translational roadmap that follow biomarker discovery.^14^ Moreover, very few studies explored the effectiveness of the proposed biomarkers in prediagnostic samples collected from asymptomatic patients, which is essential for determining how early in the latency period can they detect PDAC.^8^, ^15^, ^16^, ^17^, ^18^, ^19^, ^20^, ^21^, ^22^, ^23^, ^24^, ^25^, ^26^, ^27^ The heterogeneous study populations and analytic platforms, as well as the lack of independent validation limit evaluation on how much value any of these biomarkers hold for earlier PDAC detection. So far, only two studies reported on biomarkers in prediagnostic urine samples. In both, the Shanghai Women's Health Study (SWHS)^28^ and the Shanghai Men's Health Study (SMHS)^29^ were utilised, where Zhao et al^30^ and Cui et al,^31^ established that higher levels of urinary prostaglandin E_2_ metabolite (PGE2‐M) was associated with risk of developing PDAC, but its predictive performance was not reported.

We have previously reported on three protein biomarkers in urine, LYVE1, REG1B and TFF1,^12^, ^32^ which when combined, form a powerful panel to detect the resectable, stage I and II PDAC with both sensitivities (SN) and specificities (SP) >85%. Moreover, plasma CA19‐9 enhanced the performance of the panel (AUC = 0.992 [95% confidence interval, CI: 0.983‐1], SN = 0.963 [95% CI: 0.913‐1] and SP = 0.967 [95% CI: 0.924‐1]) in distinguishing healthy controls from stage I‐II PDAC cases.^32^ We have also developed the associated PancRISK score, an algorithm based on the protein panel, urine creatinine and age, a risk stratification tool with a binary output for risk of developing PDAC (“elevated” or “average”).^33^ While the obtained results are encouraging and clinical validation of the urinary biomarkers is ongoing (UroPanc trial, https://clinicaltrials.gov/ct2/show/NCT04449406), we aimed here to evaluate the predictive performance of PancRISK using urine samples and epidemiological data collected up to 5 years before PDAC diagnosis was established. The known lifestyle risk factors for PDAC, including smoking, obesity and heavy alcohol use are well known to increase the risk of PDAC.^34^, ^35^, ^36^, ^37^, ^38^, ^39^ Current smoking is associated with a nearly 2‐fold risk of developing PDAC^34^, ^38^ and BMI of >30 kg/m^2^ is associated with a 1.5‐fold risk of PDAC.^34^ Heavy drinking of greater than four drinks/day is associated with a 1.6‐fold risk of PDAC.^40^ Furthermore, metabolic and digestive changes are also harbingers of undiagnosed PDAC, and collectively they can serve as early indicators of PDAC as demonstrated in pooled and meta‐analyses.^36^, ^39^, ^41^, ^42^ We, therefore, also explored the performance of the urine panel in conjunction with the above risk factors for PDAC.

## 2 METHODS

### 2.1 Case and control samples selection

Samples for our study were selected from three large population‐based, prospective cohort studies, the Shanghai Women's Health Study (SWHS), the Shanghai Men's Health Studies (SMHS) and the Southern Community Cohort Study (SCCS). SWHS enrolled 75 221 women aged 40 to 70 years among those residing in seven urban districts in Shanghai, China, between December 1996 and May 2000.^28^ SMHS enrolled 61 480 men aged 40 to 74 years who were residing in eight urban communities in Shanghai, China between January 2002 and June 2006.^29^ SCCS enrolled 84 797 men and women aged 40 to 79 years, including 55 362 African Americans, from March 2002 to September 2009 from multiple communities across the 12‐state area of Southeast US.^43^ In addition to paired urine and blood specimens collected from each participant, all three cohort studies benefit from rich associated data on demographic factors, diet, lifestyle habits (eg, smoking, alcohol) and medical history, including diabetes status and its duration. All this information was collected at the time of patients' enrolment.

From each cohort, incident PDAC cases from which urine and blood samples were collected within 5 years prior to PDAC diagnosis were selected: 25 cases from SWHS, 52 from SMHS and 22 from SCCS. For each case, two controls were randomly selected and individually matched on index cases, by age at the time of urine collection (within 2 years), date of biospecimen collection (within 60 days of collection), time of sample collection (morning or afternoon), menopausal status at sample collection (in women), time since last meal (within 2 hours), sex, antibiotic use in the past week (yes/no), as well as race and year of recruitment (SCCS only).

### 2.2 Urine biomarkers and plasma CA19‐9 measurements

Commercially sourced ELISA kits were used for assaying the three biomarkers, according to the manufacturer's instructions: R&D Systems, Bio‐Techne was used for both TFF1 (Cat# DY5237) and LYVE1 (Cat# DY2089) with DuoSet Ancillary Reagent kit 2 (Cat# DY008). Urine samples were diluted 1:10 and 1:75 for TFF1 and LYVE1 ELISA, respectively. REG1B was assayed with the ELISA Pair Set, Sino Biological Inc. (Cat# SEK11638; 1:500 urine dilutions) with TMB Substrate reagent and Stop Solution from BioLegend (Cat# 421101 and 423 001).

Optical density was determined using the FLUOstar OMEGA Microplate Reader at 450 nm. Each sample was assayed in duplicate, and further repeats were run when there was a discrepancy between the duplicates. Plasma CA19‐9 was measured by Luminex (xMap technology). Urine creatinine was measured at the Clinical Biochemistry Laboratory of the University of Westminster using an ILab Aries analyser from Instrumentation Laboratory according to the manufacturer's protocol (limit of detection: 0.6 mmol/L). All the assays were performed by the research staff blinded to the sample diagnosis.

### 2.3 Statistical analysis

All protein concentration data were natural‐log‐transformed and mean‐centred prior to the analysis. The biomarker panel and affiliated PancRISK score were investigated for its ability to discriminate between cases at 1 to 5 years from diagnosis and control specimens using a receiver operating characteristics (ROC) curve approach. Given the limited number of samples no splitting of the data could be performed and the validation was done using leave‐one‐out cross‐validation. Confidence intervals (95% CIs) for AUCs were derived based on DeLong's asymptotically exact method to evaluate the uncertainty of an AUC^44^; SN and SP and 95% CI were derived using nonparametric stratified resampling with the percentile method (2000 bootstrap replicates). Logistic regression was applied to the panel, CA19‐9 and to their combination with BMI (as continuous variable) and diabetes (yes/no). All analyses were performed in R version 3.5.1 (R Foundation for Statistical Computing; http://www.r-project.org/foundation/) using the ROCR and pROC packages. The *P*‐values were calculated with nonparametric Mann‐Whitney *t*‐test for continuous variables and with Fisher's exact test for categorical variables using GraphPad PRISM v9.

For multivariate analysis, the following covariates assessed at the time of sample collection were included: smoking history (never/former/current), alcohol consumption (heavy drinking defined as >4 drinks/day for men and >3 drinks/day for women, categorised based on quantity during the period of drinking; all other drinking levels categorised as moderate), BMI (kg/m^2^, as continuous variable), diabetes status (no, yes ≤3 years and >3 years of sample collection, as an ordinal variable), history of any familial cancer (no/yes; yes included first degree relatives), asthma (no/yes), hypertension (no/yes) and education (less than high school/high school/vocational training/college graduate).

To compare the levels of the three biomarkers between cases and controls, control samples were categorised into four groups according to different percentiles of each of the three urine biomarkers adjusted for urine creatinine levels. ANCOVA analysis adjusted for age at sample collection and post hoc test was applied.

### 2.4 Results

Demographic and epidemiological data of 99 cases and 198 controls are summarised in Table 1. Except for the significant difference in smoking habits observed between cases and controls in the SCCS cohort (current smokers 72% in cases, *P* = .0177, 35% in controls, *P* = .0372) all the other characteristics in both the SCCS and the two Shanghai cohorts did not significantly differ between the cases and controls (Table 1).

**TABLE 1:** Demographic details of the controls and cases across the cohort studies

	SCCS control (n = 44)	SCCS case (n = 22)	SWHS control (n = 50)	SWHS cases (n = 25)	SMHS control (n = 104)	SMHS cases (n = 52)
*Age, Median (IQR) (years)*	57 (51‐62)	58 (52‐60)	64.91 (58.19‐67.31)	64.77 (57.71‐68)	66.97 (57.23‐71.91)	66.82 (57.05‐71.14)
*Gender*						
Female	16 (36%)	8 (36%)	50 (100%)	25 (100%)	0 (0%)	0 (0%)
Male	28 (64%)	14 (64%)	0 (0%)	0 (0%)	104 (100%)	52 (100%)
*Diabetes at enrolment*						
Diabetes ≤3 years	4 (9.1%)	2 (0%)	0 (0%)	3 (12%)	3 (2.9%)	3 (5.8%)
Diabetes >3 years	10 (22.7%)	7 (40%)	4 (8%)	0 (0%)	4 (3.8%)	8 (15.4%)
No diabetes	30 (68.2%)	13 (59.2%)	46 (92%)	22 (88%)	97 (93.3%)	41 (78.8%)
*Familial cancer*						
First degree	23 (52.3%)	12 (54.5%)	14 (28%)	9 (36%)	30 (28.8%)	13 (25%)
No FC	21 (47.7%)	10 (45.6%)	36 (72%)	16 (64%)	74 (71.2%)	39 (75%)
*Smoking*						
Current	17 (39.5%)	16 (72%)^a^	2 (4%)	0 (0%)	47 (45.2%)	25 (48%)
Former	15 (34.9%)	2 (9%)^b^	1 (2%)	0 (0%)	16 (15.4%)	7 (13.5%)
Never	11 (25.6%)	4 (18%)	47 (94%)	0 (0%)	41 (39.4%)	20 (38.5%)
*Unknown*			*1*			
*Drinking*						
Heavy	6 (15.4%)	5 (29.4%)	0 (0%)	0 (0%)	5 (4.9%)	2 (3.85%)
Moderate	16 (41%)	6 (35.3%)	1 (2%)	0 (0%)	30 (29.1%)	12 (23%)
Never	17 (43.6%)	6 (35.3%)	48 (98%)	0 (0%)	68 (66%)	38 (73%)
*Unknown*	*5*	*5*	*1*		*1*	
*BMI enrolment, median (IQR)*	29.7 (26.2‐34.1)	29.19 (25.23‐31.84)	24.2 (21.9‐26.8)	22.58 (21.01‐25.62)	23.8 (21.4‐25.9)	23.27 (21.41‐25.01)
*BMI peak, median (IQR)*	31.8 (27.9‐37.5)	33.6 (29.39‐39.33)	24.4 (22.2‐27.8)	23.23 (21.38‐26.08)	23.8 (21.8‐25.9)	23.635 (21.76‐25.12)
*Hypertension*						
Yes	27 (61.4%)	15 (68.2%)	22 (44%)	11 (44%)	36 (34.6%)	17 (32.7%)
No	17 (38.6%)	7 (31.8%)	28 (56%)	14 (56%)	68 (65.4%)	35 (67.3%)
*Asthma*						
Yes	8 (18.2%)	5 (22.7%)	0 (0%)	3 (12%)	2 (1.9%)	0 (0%)
No	36 (81.8%)	17 (77.3%)	50 (100%)	22 (88%)	102 (98.1%)	52 (100%)
*Education*						
Less than high school	13 (29.5%)	5 (22.7%)	34 (68%)	18 (72%)	54 (51.9%)	23 (44.2%)
High school	17 (38.6%)	7 (31.8%)	9 (18%)	6 (24%)	32 (30.8%)	15 (28.8%)
Vocational training/college	9 (20.5%)	6 (27.5%)	4 (8%)	0 (0%)	6 (5.8%)	10 (19%)
College graduate	5 (11.4%)	4 (18%)	3 (6%)	1 (4%)	12 (11.5%)	4 (7.7%)
*Cancer stage at diagnosis*						
I		2 (11%)		3 (17.6%)		2 (9%)
II		3 (16%)		7 (41.2%)		5 (22.7%)
III		2 (11%)		1 (5.8%)		1 (4.5%)
IV		12 (63%)		6 (35.3%)		14 (63.6%)
*Unknown*		*3*		*8*		*30*
*Deceased*		22 (100%)		23 (92%)		50 (96%)
*Survival, median (IQR) (months)*		3.5 (1‐8)		7.68 (3.36‐17.34)		6.18 (2.34‐11.76)
*Time group (years)*						
0‐1		4		6		8
1‐2		2		6		12
2‐3		6		5		10
3‐4		4		4		15
4‐5		6		4		7

*Note*: BMI was measured in kg/m^2^.

Abbreviations: FC, familial cancer; IQR, Inter Quartile Range.

^a^ *P* = .0177.

^b^ *P* = .0372.

The expression of three urinary biomarkers, urine creatinine levels and plasma CA19‐9 values are provided in Table S1. Intra‐ and interassay coefficient of variation (CV) for LYVE1, TFF1 and REG1B, were 26%, 15%, 11% and 18%, 26%, 24%, respectively. Univariate analysis showed a statistically significant increase of urinary REG1B up to 12 and 24 months before PDAC diagnosis as compared to controls (*P* < .001 and *P* = .0022, respectively, Wilcoxon test) and urinary LYVE1 up to 12 months before PDAC diagnosis (*P* < .05, Wilcoxon test). Increase in TFF1 expression did not reach statistical significance (Table 2). This is due to high levels of TFF1 expression in controls, especially in SCCS cohort, which was almost two to three times the values seen in SMHS and SWHS, respectively (*P* = .008; Table S2).

**TABLE 2:** Wilcoxon rank‐sum test (the shown *P*‐values were obtained testing each year group against the combined controls [n = 198])

	0‐1 year (n = 18)	0‐2 years (n = 38)	0‐3 years (n = 59)	0‐4 years (n = 82)	0‐5 years (n = 99)
LYVE1	0.013	0.046	0.216	0.245	0.104
REG1B	<0.001	0.002	0.106	0.252	0.252
TFF1	0.15	0.273	0.847	0.613	0.466
Creatinine	0.161	0.274	0.598	0.999	0.949
Age	0.404	0.877	0.374	0.578	0.862

It was also observed that some of the control values for all three biomarkers were higher than in the cases. To see if this was due to any known confounders (fasting, sample collection time, therapies taken) or any of the associated comorbidities, we have compared their distribution (Table S3, Group 1). None of these confounding factors were shown to have an effect; importantly, the presence of up to five or more comorbidities (including coronary heart disease [CHD] or acute myocardial infarction [AMI], diabetes, cancer [in SWHS only], stroke, hepatitis, chronic pulmonary disease and gastrointestinal ulcer disease) did not show any significant effect on the biomarker levels. The same was true also for the cases (Table S3, Group 2).

**FIGURE 1:**
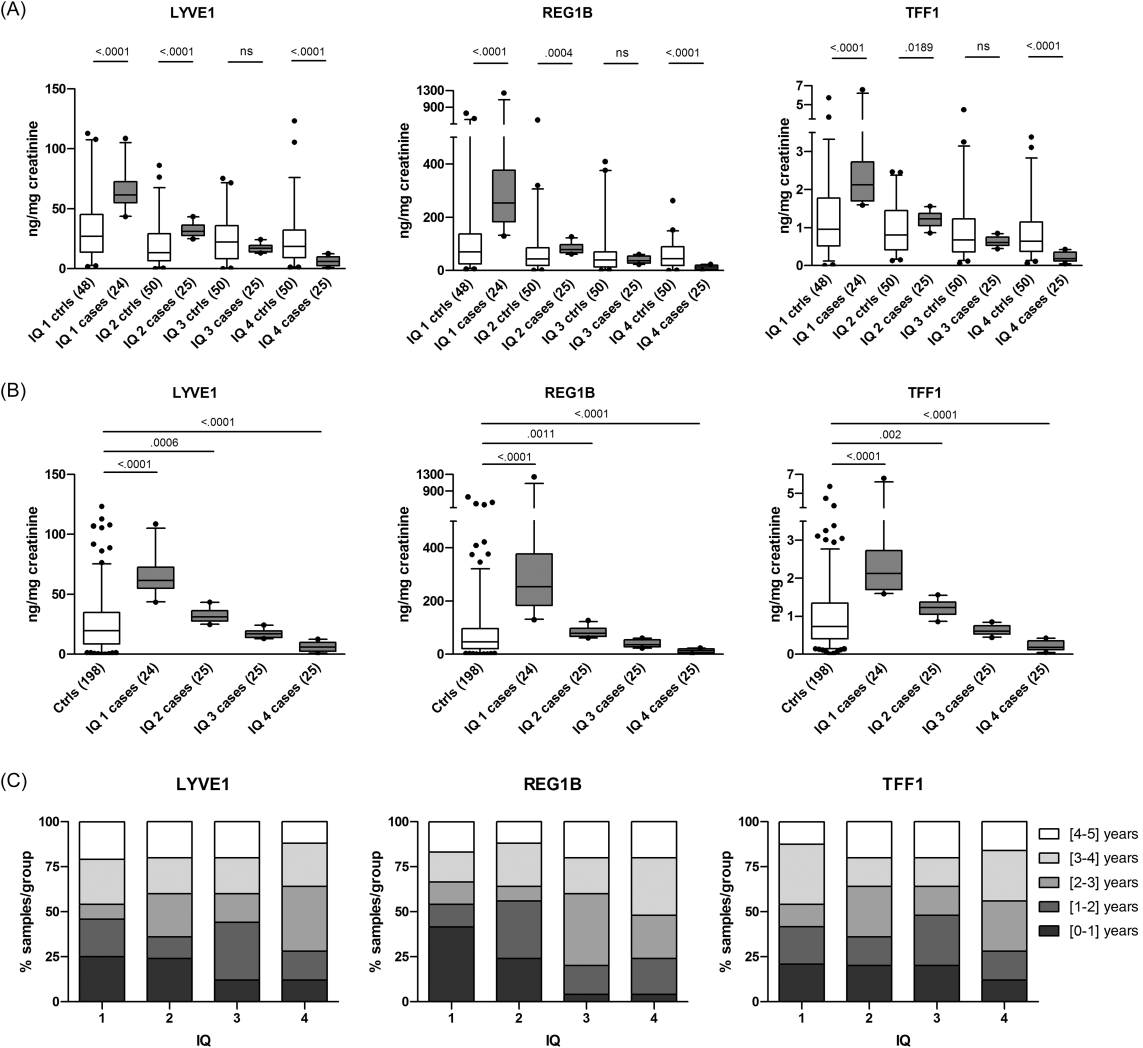
Performance of the biomarkers. (A) Mann‐Whitney *t*‐test between cases in IQ1, IQ2, IQ3, IQ4 and respective matched controls (the number of samples in each group is indicated in the brackets; ns, not significant); (B) Mann‐Whitney *t*‐test between cases in IQ1, IQ2, IQ3, IQ4 and the combined controls; (box and whisker histograms 5‐95 percentile); (C) distribution of cases (%) by year group in each IQ

The interquartile analysis of the cases and controls was performed next (Figure 1A‐C). In cases, the upper and second interquartile (IQ1 and IQ2) of each biomarker (LYVE1, TFF1 and REG1B) were significantly higher than the matched controls. The third (IQ3) of cases was not significantly different from the matched controls, and the lowest (IQ4) of cases was significantly lower than the matched controls (Figure 1A). The same pattern was also observed when all the controls were combined (Figure 1B). When looking at the distribution of the cases by year group in each IQ, it was evident that IQ1 consisted mostly of cases in the 0‐ to 2‐year group (45%, 54% and 42% for LYVE1, REG1B and TFF1, respectively; Figure 1C).

**FIGURE 2:**
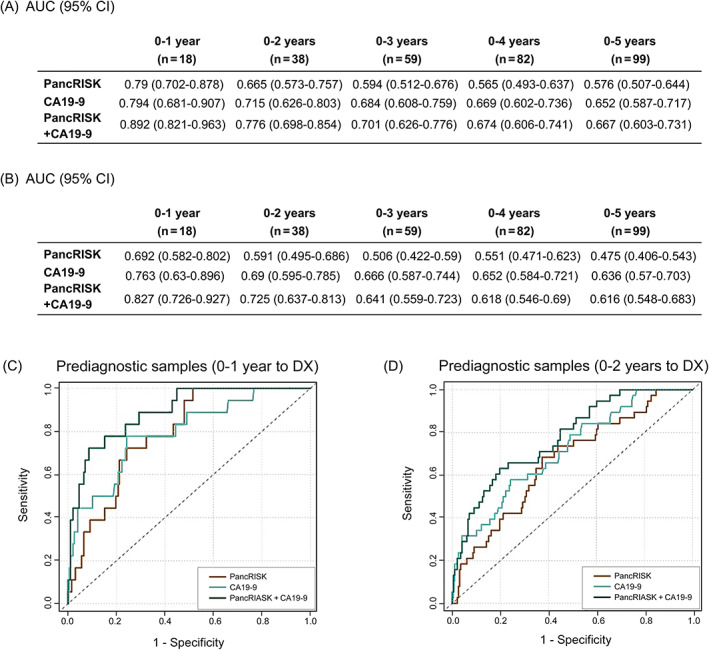
Performance of the PancRISK alone and in combination with plasma CA19‐9. (A) AUC (no split, training and testing on the same data) and (B) AUC, leave‐one‐out cross‐validation up to 5 years prior to diagnosis. Receiver operating characteristic (ROC) curves of the PancRISK and CA19‐9 alone and in combination was used to evaluate their performance in distinguishing prediagnostic cases (C) 1 year and (D) 2 years before diagnosis (DX)

As the three biomarkers perform best when they are combined,^12^, ^32^ they were next assessed as the panel, together with creatinine and age, with the PancRISK algorithm.^33^ We analysed the performance of the panel alone or with plasma CA19‐9 in discriminating between prediagnostic cases and control urine samples. The PancRISK alone resulted in AUCs of 0.79 (95% CI: 0.702‐0.878) and 0.665 (95% CI: 0.573‐0.757) for PDAC diagnosed up to 12 and 24 months, respectively, before PDAC diagnosis (Figure 2A) and AUCs of 0.692 (95% CI: 0.982‐0.802) and 0.591 (95% CI: 0.495‐0.686) in the leave‐one‐out validation (Figure 2B). The discriminatory power of CA19‐9 was similar to the PancRISK, but their combination enhanced the performance with AUCs to 0.892 (95% CI: 0.821‐0.963) and 0.776 (95% CI: 0.698‐0.854) 12 and 24 months before cancer diagnosis (Figure 2A). In cross‐validation, the combined PancRISK and CA19‐9 panel showed good discrimination of cases and controls (AUC = 0.827, 95% CI: 0.726, 0.927 for PDAC diagnosed up to 12 months, and AUC = 0.725, 95% CI: 0.637, 0.813 for PDAC diagnosed up to 24 months; Figure 2B). ROC curves for panel of biomarkers and CA19‐9 alone and their combination are shown in Figure 2C,D for the 0‐ to 1‐year and 0‐ to 2‐year groups, respectively.

Table 3 provides SN and SP values for PancRISK alone or in combination with CA19‐9. At SP fixed at 90%, the SN was 72.2% (Table 3A), and at fixed SN of 90%, the SP was 59.6% (Table 3B) 1 year prior to diagnosis. For samples taken within 2 years before PDAC diagnosis, the performance of combined urinary panel and CA19‐9 was 60% SN at fixed 80% SP and 53% SP with 80% SN.

**TABLE 3:** Specificity and sensitivity of PancRISK and plasma CA19‐9, alone and in combination—(A) Sensitivity (SN) at fixed specificity (SP) for PancRISK (i), CA19‐9 (ii) and combined PancRISK and CA19‐9; (B) SP at fixed SN for PancRISK (i), CA19‐9 (ii) and combined PancRISK and CA19‐9

(A) Sensitivity (95% CI) at fixed specificity					
Specificity	0‐1 year	0‐2 years	0‐3 years	0‐4 years	0‐5 years
(i) PancRISK: Sensitivity (95% CI) at fixed specificity					
0.75	0.667	0.421	0.339	0.342	0.364
0.8	0.5	0.368	0.254	0.256	0.242
0.85	0.444	0.29	0.22	0.171	0.162
0.9	0.389	0.237	0.17	0.073	0.111
0.95	0.167	0.184	0.102	0.012	0.02
(ii) CA19‐9: Sensitivity (95% CI) at fixed specificity					
0.75	0.722	0.553	0.475	0.439	0.448
0.8	0.589	0.459	0.373	0.343	0.333
0.85	0.5	0.368	0.322	0.268	0.263
0.9	0.472	0.342	0.271	0.22	0.195
0.95	0.389	0.29	0.22	0.171	0.141
(iii) PancRISK + CA19‐9: Sensitivity (95% CI) at fixed specificity					
0.75	0.833	0.658	0.542	0.476	0.475
0.8	0.778	0.605	0.458	0.402	0.384
0.85	0.778	0.54	0.373	0.305	0.283
0.9	0.722	0.447	0.305	0.244	0.202
0.95	0.5	0.29	0.237	0.171	0.141

(B) Specificity (95% CI) at fixed sensitivity					
Sensitivity	0‐1 year	0‐2 years	0‐3 years	0‐4 years	0‐5 years
(i) PancRISK: Specificity (95% CI) at fixed sensitivity					
0.75	0.687	0.49	0.394	0.303	0.349
0.8	0.586	0.399	0.328	0.263	0.268
0.85	0.546	0.303	0.197	0.207	0.217
0.9	0.52	0.212	0.141	0.167	0.141
0.95	0.5	0.177	0.111	0.126	0.086
(ii) CA19‐9: Specificity (95% CI) at fixed sensitivity					
0.75	0.742	0.515	0.52	0.51	0.489
0.8	0.576	0.475	0.479	0.461	0.421
0.85	0.515	0.373	0.373	0.401	0.333
0.9	0.358	0.302	0.273	0.315	0.258
0.95	0.263	0.253	0.194	0.227	0.151
(iii) PancRISK + CA19‐9: Specificity (95% CI) at fixed sensitivity					
0.75	0.854	0.576	0.47	0.475	0.53
0.8	0.773	0.535	0.444	0.45	0.399
0.85	0.712	0.48	0.381	0.384	0.338
0.9	0.596	0.424	0.318	0.333	0.278
0.95	0.561	0.359	0.242	0.268	0.202

The distribution of urine biomarkers across various covariates is shown Table S4. Except difference in biomarker values by asthma status in the SCCS cohort (*P* = .002), no other factors influenced levels of urine biomarkers.

When the variables were analysed by categories in pooled controls and cases by year groups, BMI was statistically different in the prediagnostic group 0 to 1 year (*P* = .027) and 0 to 2 years prior to PDAC diagnosis (*P* = .04; Table S5). The analysis of diabetes showed that there is a statistically significant difference between the presence of diabetes in cases only when all the cases were combined (0‐5 years before PDAC diagnosis; *P* = .03; Table S5) likely due to limited number of samples per year. The length of diabetes information that was available was calculated from the date of collection, as well as from the date of cancer diagnosis. All cases with diabetes, except one with diabetes diagnosed within 3 years from cancer diagnosis, had long‐standing diabetes, which precluded any analysis of biomarkers and new onset diabetes. To assess whether BMI and diabetes were associated with cancer diagnosis, we performed the analysis adjusted by study site as members of the SCCS cohort were mostly overweight and nearly 35% of them were diabetic, compared to <10% among the Shanghai cohort. Significantly lower BMI was seen in the patients who were diagnosed 0‐ to 1‐year after study enrolment (OR = 0.843, 95% CI: 0.724‐0.964; *P* = .019), and borderline in cases diagnosed 0‐ to 2‐years after enrolment (OR = 0.911; 95% CI: 0.823‐1; *P* = .059). For diabetes, only when all cases and controls were analysed, a positive association with PDAC diagnosis was seen (OR = 2.236; 95% CI: 1.156‐4.337; *P* = .017).

Next, we assessed if adding BMI value and diabetes information (yes/no) to PancRISK would further improve its performance (Table 4). Interestingly, addition of BMI and diabetes information generally improved model performance up to 2 years before PDAC diagnosis, although diabetes resulted in only modest AUC increase (Table 4A). In leave‐one‐out‐validation, combination of PancRISK, CA19‐9 and BMI performed best (AUC = 0.733) for predicting PDAC diagnosed within 2 years; however, the AUC increase after adding BMI was not significant (*P* = .711; Table 4B).

**TABLE 4:** Performance of the PancRISK in combination with plasma CA19‐9, BMI and Diabetes—Receiver operating characteristic (ROC) curves of the PancRISK and CA19‐9, BMI and Diabetes alone and in combination was used to evaluate their performance in distinguishing prediagnostic cases up to 5 years before diagnosis

(A) AUC (no split, training and testing on the same data)					
	0‐1 year	0‐2 years	0‐3 years	0‐4 years	0‐5 years
PancRISK	0.79 (0.702‐0.878)	0.665 (0.573‐0.757)	0.594 (0.512‐0.676)	0.565 (0.493‐0.637)	0.576 (0.507‐0.644)
PancRISK + BMI	0.804 (0.71‐0.898)	0.68 (0.591‐0.768)	0.602 (0.522‐0.681)	0.585 (0.515‐0.656)	0.575 (0.507‐0.643)
PancRISK + Diabetes	0.807 (0.726‐0.888)	0.68 (0.587‐0.773)	0.606 (0.52‐0.691)	0.57 (0.495‐0.646)	0.605 (0.536‐0.674)
PancRISK + BMI + Diabetes	0.829 (0.745‐0.913)	0.701 (0.61‐0.793)	0.61 (0.526‐0.694)	0.594 (0.521‐0.667)	0.587 (0.518‐0.656)
CA19‐9	0.794 (0.681‐0.907)	0.715 (0.626‐0.803)	0.684 (0.608‐0.759)	0.669 (0.602‐0.736)	0.652 (0.587‐0.717)
PancRISK + CA19‐9	0.892 (0.821‐0.963)	0.776 (0.698‐0.854)	0.701 (0.626‐0.776)	0.674 (0.606‐0.741)	0.667 (0.603‐0.731)
PancRISK + CA19‐9 + BMI	0.911 (0.852‐0.971)	0.79 (0.713‐0.868)	0.706 (0.63‐0.782)	0.681 (0.613‐0.748)	0.667 (0.602‐0.732)
PancRISK + CA19‐9 + Diabetes	0.89 (0.815‐0.965)	0.778 (0.7‐0.857)	0.701 (0.626‐0.776)	0.679 (0.612‐0.746)	0.685 (0.622‐0.749)
PancRISK + CA19‐9 + BMI + Diabetes	0.915 (0.86‐0.97)	0.793 (0.715‐0.87)	0.712 (0.636‐0.788)	0.69 (0.623‐0.757)	0.677 (0.612‐0.742)

(B) AUC, leave‐one‐out cross‐validation					
	0‐1 year	0‐2 years	0‐3 years	0‐4 years	0‐5 years
PancRISK	0.692 (0.582‐0.802)	0.591 (0.495‐0.686)	0.506 (0.422‐0.59)	0.551 (0.479‐0.623)	0.475 (0.406‐0.543)
PancRISK + BMI	0.688 (0.562‐0.814)	0.589 (0.492‐0.685)	0.507 (0.425‐0.589)	0.489 (0.417‐0.56)	0.539 (0.471‐0.607)
PancRISK + Diabetes	0.711 (0.608‐0.815)	0.606 (0.509‐0.704)	0.496 (0.407‐0.585)	0.534 (0.457‐0.61)	0.517 (0.446‐0.588)
PancRISK + BMI + Diabetes	0.711 (0.597‐0.825)	0.618 (0.52‐0.715)	0.487 (0.398‐0.576)	0.507 (0.431‐0.582)	0.501 (0.429‐0.573)
CA19‐9	0.763 (0.63‐0.896)	0.69 (0.595‐0.785)	0.666 (0.587‐0.744)	0.652 (0.584‐0.721)	0.636 (0.57‐0.703)
PancRISK + CA19‐9	0.827 (0.726‐0.927)	0.725 (0.637‐0.813)	0.641 (0.559‐0.723)	0.618 (0.546‐0.69)	0.616 (0.548‐0.683)
PancRISK + CA19‐9 + BMI	0.834 (0.735‐0.932)	0.733 (0.643‐0.822)	0.644 (0.561‐0.728)	0.627 (0.554‐0.7)	0.607 (0.538‐0.677)
PancRISK + CA19‐9 + Diabetes	0.82 (0.716‐0.925)	0.717 (0.629‐0.806)	0.637 (0.555‐0.719)	0.615 (0.542‐0.687)	0.628 (0.561‐0.696)
PancRISK + CA19‐9 + BMI + Diabetes	0.82 (0.716‐0.925)	0.73 (0.64‐0.819)	0.643 (0.56‐0.726)	0.632 (0.56‐0.704)	0.616 (0.546‐0.685)

## 3 DISCUSSION

In this nested case‐control study, we assessed the performance of our urine biomarker panel in prediagnostic samples, and demonstrated that it can distinguish cases from controls up to 2 years prior to clinical diagnosis of PDAC. We opted to use nested case‐control approach, rather than case‐cohort design, as it allows for tighter matching on many factors that may affect biomarker levels. We applied the internal validation approach and we reported on the validated AUC estimators.

We also confirmed a good complementarity of our urinary panel to plasma CA19‐9, as already reported.^12^, ^32^ Several previous studies similarly combined single or multibiomarker panels in serum or plasma with CA19‐9 to improve their performance, and in general, these studies were also performed using limited number of prediagnostic samples.^8^, ^16^, ^17^, ^19^, ^20^, ^21^, ^22^, ^25^ Where provided, obtained SNs were comparable or lower than ours. For example, in a study by Nolen et al,^19^ who interrogated 70 prediagnostic sera from the prospective Prostate, Lung, Colorectal and Ovarian Cancer Screening (PLCO) cohort, AUC of 0.69 and SN of 32% at SP of 95% were obtained for CA19‐9 in combination with CEA and Cyfra 21‐1 up to 1 year before PDAC diagnosis. In comparison, the AUC for our urinary panel combined with CA19‐9 was 0.89, with SN of 50% at SP of 95%. Likewise, Honda et al^25^ assessed the performance for the combination of CA19‐9 and ApoA2 isoform in plasma samples from the European Prospective Investigation into Cancer and Nutrition (EPIC) cohort collected up to 18 months prior to PDAC diagnosis, obtaining AUC of 0.75, with SN 43% at SP 98%.^25^ Two further studies provide only the AUC values^16^, ^22^: in Faca et al,^16^ AUC of 0.91 was obtained for the combination of TIMP1, IGFBP4, LCN2, REG3, REG1A and CA19‐9 in 13 samples collected 7 to 13 months prior to PDAC diagnosis and part of the Carotene and Retinol Efficacy Trial (CARET) study. Jenkinson et al,^22^ reported AUC of 0.85 for TSP‐1 plus CA19‐9 in 64 samples from the UK Collaborative Trial of Ovarian Cancer Screening (UKCTOCS) collected up to 2 years prior to PDAC diagnosis. Regarding the serum CA19‐9, O'Brien et al^21^ demonstrated that this commonly used biomarker can be detected in prediagnostic PDAC samples at 95% SP, with a SN of 68% up to 1 year and 53% up to 2 years prior to PDAC diagnosis.^21^ These values are in general much higher than observed in our and in other studies,^19^, ^25^ and could potentially be explained by differences in sample numbers, and interrogation of different cohorts.

While the obtained performance of our biomarkers in the tested prediagnostic specimens is encouraging, there were a number of limitations that hampered our analysis. Namely, the number of cohorts that collected prediagnostic urine samples is exceedingly small, and the number of PDAC cases in the cohorts analysed here was scarce despite their large size (>70 000 enrolled in each cohort). This limits the confidence in our AUC estimates and could have imposed greater sampling error. The low prevalence of PDAC cases both in general and in such invaluable cohorts is unfortunately a commonly encountered problem. To overcome this issue, we have combined three different cohorts, although we have noticed that the levels of our biomarkers in controls varied by Institution and was particularly high in the SCCS cohort (even higher than obtained in samples from patients with benign hepatobiliary diseases in our previous analyses^12^, ^32^). As analyses of comorbidities did not appear to have any effect on the biomarker levels, the difference in their values could potentially be driven by geographic origin or environmental cues, which should be explored in the future.

Finally, the samples utilised in our study were collected 13 to 25 years before this analysis. While we proved that our biomarkers do not show significant daily variation, and that they are stable for up to 5 days at room temperature,^32^ we cannot at present rule out the effect of long storage on our three proteins that could potentially also explain the high intra‐ and interassay CVs observed in the ELISA data. However, this problem will not be encountered in any envisaged clinical use of our biomarker panel.

The association of BMI and diabetes with PDAC was shown previously.^36^, ^39^ Using the same Shanghai cohorts as us, Zhao et al^30^ and Cui et al^31^ established that individuals with high level of urinary prostaglandin E_2_ metabolites (PGE‐M) had an increased risk of developing PDAC, with ORs from 1.63 for the second quartile up to 1.94 for the upper quartile. Interestingly, this positive association was more evident among those who had BMI <25 kg/m^2^ ^31^ and among subjects with diabetes history.^30^ We assessed if adding BMI and diabetes to PancRISK would further improve its performance and show that combination with CA19‐9 and BMI resulted in the overall improvement in PancRISK performance. This important finding will now need to be validated in an independent set of larger number of samples.

In summary, we have shown that our urinary biomarker panel in combination with CA19‐9 can distinguish prediagnostic cases from controls at least up to 2 years before the diagnosis of PDAC and that this performance can potentially be further improved by adding epidemiological and clinical data, in particular BMI. We have also demonstrated that common comorbidities do not act as confounders, reinforcing thus the confidence in our biomarkers. Combined, these data provide a first glimpse of potential utility of our urine biomarkers for earlier detection of latent PDAC before the occurrence of clinical symptoms.

These results strengthen the confidence of the urine panel and affiliated PancRISK, which are intended for surveillance in high‐risk groups as well as detection of sporadic PDAC in patients with nonspecific, but suggestive of PDAC symptoms. The urine panel and affiliated PancRISK are currently being validated in a prospective clinical study (UroPanc) with the aim of implementing them in clinical practice as noninvasive stratification tool for selection of patients that require further, invasive and expensive clinical workup, which will likely significantly improve the current diagnostic pathway for such patients.

## AUTHOR CONTRIBUTIONS

The work reported in the article has been performed by the authors, unless clearly specified in the text. Silvana Debernardi: Conceptualization; Data curation; Formal Analysis; Investigation; Methodology; Validation; Visualisation; Writing—original draft; Writing—review & editing. Oleg Blyuss: Formal Analysis; Visualisation; Writing—review & editing. Daria Rycyk: Investigation; Methodology; Validation; Writing—review & editing. Kirtiman Srivastava: Investigation; Methodology; Validation; Writing—review & editing. Christie Y. Jeon: Conceptualization; Data curation; Investigation; Project administration; Resources; Writing—review & editing. Hui Cai: Formal Analysis; Writing—review & editing. Qiuyin Cai: Methodology; Writing—review & editing. Xiao‐Ou Shu: Conceptualization; Data curation; Project administration; Resources; Writing—review & editing. Tatjana Crnogorac‐Jurcevic: Conceptualization; Data curation; Funding acquisition; Investigation; Project administration; Supervision; Writing—original draft; Writing—review & editing.

## FUNDING INFORMATION

This work was funded by Cancer Research UK Early Detection and Diagnosis project grant (reference number A26239) and by grants from the National Institute of Health (UM1CA173640, UM1CA182910 and U01CA202979).

## CONFLICT OF INTEREST

The authors have no conflict of interest to declare.

## ETHICS STATEMENT

The study was performed with Ethics approval from the participating Centres. Informed written consent was obtained from each participant for the retaining of the epidemiological information obtained through interviews and for the collection of blood and urine specimens.

## Supporting information


**Table S1** Measurement of plasma Ca19‐9, creatinine and the three urinary biomarkers, LYVE1, TFF1 and REG1B.
**Table S2**. Statistical differences in controls between SCCS and SM/WHS cohorts.
**Table S3**. Distribution of possible confounders by four groups for each urinary biomarker. Group 1: Controls with biomarker ≥ upper quartiles of controls; Group 2: Cases with biomarker ≥ upper quartiles of controls; Group 3: Controls with biomarker < upper quartiles of controls; Group 4: Cases with biomarker < upper quartiles of controls.
**Table S4**. Covariate analysis.

## Data Availability

The data that support the findings of our study are available from the corresponding author upon reasonable request.
