# Supplementary material for: Urine biomarkers enable pancreatic cancer detection up to 2 years before diagnosis
Source: Int J Cancer. 2022 Sep 23;152(4):769–80. doi: 10.1002/ijc.34287 (PMC9789171; doi:10.1002/ijc.34287)
Supplement: Supplementary file 1 — Table S1 Measurement of plasma Ca19‐9, creatinine and the three urinary biomarkers, LYVE1, TFF1 and REG1B. Table S2. Statistical differences in controls between SCCS and SM/WHS cohorts. Table S3. Distribution of possible confounders by four groups for each urinary biomarker. Group 1: Controls with biomarker ≥ upper quartiles of controls; Group 2: Cases with biomarker ≥ upper quartiles of controls; Group 3: Controls with biomarker < upper quartiles of controls; Group 4: Cases with biomarker < upper quartiles of controls. Table S4. Covariate analysis. [file IJC-152-769-s001.pdf]

Urine biomarkers enable pancreatic cancer detection up to 2 years before diagnosis

Silvana Debernardi, Oleg Blyuss, Daria Rycyk, Kirtiman Srivastava, Christie Y Jeon, Hui Cai, Qiuyin Cai, Xiao-Ou Shu, Tatjana Crnogorac-Jurcevic

Supplementary Tables

Supporting Information Table S1.
Supporting Information Table S2.
Supporting Information Table S3.
Supporting Information Table S4.
Supporting Information Table S5.

Supporting Information Table S1. Measurement of plasma Ca19-9, creatinine and the three urinary biomarkers, LYVE1, TFF1 and REG1B.

STUDY	Sample ID	Cases and matched ctrls year groups	CA19-9 U/ml	Creatinine grams/L	LYVE1 ng/ml	TFF1 ng/ml	REG1B ng/ml
SWHS	**1**	[0-1] case	92.87	0.20	2.764	0.026	25.693
SWHS	**2**	[0-1] case	18.37	1.27	43.746	1.634	232.558
SWHS	**3**	[0-1] case	22.07	0.28	2.017	0.580	75.220
SWHS	**4**	[0-1] case	9.55	1.33	7.685	0.089	78.341
SWHS	**5**	[0-1] case	72.71	0.82	47.486	0.797	305.248
SWHS	**6**	[0-1] case	18.94	1.05	47.041	2.510	79.423
SMHS	**7**	[0-1] case	11.70	0.62	36.581	0.487	122.770
SMHS	**8**	[0-1] case	17.61	1.75	48.976	2.158	685.835
SMHS	**9**	[0-1] case	9.35	2.52	66.727	0.995	34.625
SMHS	**10**	[0-1] case	12.25	0.17	0.000	0.030	0.712
SMHS	**11**	[0-1] case	28.19	0.25	1.347	0.405	0.384
SMHS	**12**	[0-1] case	7.45	1.61	97.444	3.166	147.226
SMHS	**13**	[0-1] case	11.26	0.59	38.462	1.232	109.407
SMHS	**14**	[0-1] case	15.54	0.25	1.235	0.069	1.399
SCCS	**15**	[0-1] case	11.16	1.92	6.273	2.073	68.741
SCCS	**16**	[0-1] case	40.03	0.48	4.235	0.509	4.741
SCCS	**17**	[0-1] case	24.00	0.85	14.552	0.891	70.575
SCCS	**18**	[0-1] case	37.65	2.80	19.204	3.450	53.620
SWHS	**19**	[0-1] ctrl	16.76	0.28	1.543	0.032	25.190
SWHS	**20**	[0-1] ctrl	12.10	1.24	6.204	0.182	81.461
SWHS	**21**	[0-1] ctrl	15.32	0.20	1.725	0.104	57.302
SWHS	**22**	[0-1] ctrl	8.36	0.76	3.097	0.147	22.875
SWHS	**23**	[0-1] ctrl	9.55	0.85	16.458	4.858	80.153
SWHS	**24**	[0-1] ctrl	11.32	2.63	51.285	0.811	61.328
SWHS	**25**	[0-1] ctrl	8.83	1.19	43.821	0.817	204.976
SWHS	**26**	[0-1] ctrl	11.44	1.58	12.004	0.895	56.698
SWHS	**27**	[0-1] ctrl	62.38	0.28	31.939	0.429	213.763
SWHS	**28**	[0-1] ctrl	39.69	0.68	72.532	0.378	122.497
SWHS	**29**	[0-1] ctrl	5.91	1.16	82.850	3.492	692.162
SWHS	**30**	[0-1] ctrl	23.93	1.10	23.847	0.278	58.644
SMHS	**31**	[0-1] ctrl	8.36	1.50	104.788	1.916	116.553
SMHS	**32**	[0-1] ctrl	7.36	0.45	19.989	0.165	108.854
SMHS	**33**	[0-1] ctrl	10.01	0.48	7.467	0.210	47.366
SMHS	**34**	[0-1] ctrl	11.18	0.42	1.380	0.735	24.128
SMHS	**35**	[0-1] ctrl	27.82	1.64	22.195	2.172	143.121
SMHS	**36**	[0-1] ctrl	35.87	2.35	11.901	1.127	16.103
SMHS	**37**	[0-1] ctrl	26.79	0.54	20.794	0.296	21.457
SMHS	**38**	[0-1] ctrl	34.38	0.82	70.709	0.860	142.978
SMHS	**39**	[0-1] ctrl	14.43	0.37	3.729	0.167	0.275
SMHS	**40**	[0-1] ctrl	10.19	0.90	1.427	0.040	3.647
SMHS	**41**	[0-1] ctrl	10.90	4.07	72.611	1.440	341.396
SMHS	**42**	[0-1] ctrl	48.28	2.23	68.698	1.059	147.431
SMHS	**43**	[0-1] ctrl	8.66	1.10	75.231	0.612	165.115
SMHS	**44**	[0-1] ctrl	8.55	0.45	19.139	0.778	9.825
SMHS	**45**	[0-1] ctrl	12.71	0.71	37.669	1.105	38.628
SMHS	**46**	[0-1] ctrl	9.12	0.17	0.317	0.140	2.251
SCCS	**47**	[0-1] ctrl	11.43	0.71	17.544	1.531	70.575
SCCS	**48**	[0-1] ctrl	11.01	1.58	8.781	2.186	96.509
SCCS	**49**	[0-1] ctrl	23.73	0.59	6.179	0.417	23.108
SCCS	**50**	[0-1] ctrl	10.75	0.40	9.276	0.717	14.074
SCCS	**51**	[0-1] ctrl	10.75	0.45	5.043	0.089	4.955
SCCS	**52**	[0-1] ctrl	11.70	0.88	22.758	2.050	239.526

continue

SCCS	**53**	[0-1] ctrl	35.26	0.45	17.182	0.765	47.641
SCCS	**54**	[0-1] ctrl	9.78	0.57	1.538	0.620	7.569
SWHS	**55**	[1-2] case	15.04	0.96	16.438	0.170	136.323
SWHS	**56**	[1-2] case	9.30	0.71	9.793	0.123	46.128
SWHS	**57**	[1-2] case	8.95	0.82	44.168	2.107	70.373
SMHS	**58**	[1-2] case	11.96	0.54	1.529	0.311	8.393
SMHS	**59**	[1-2] case	56.49	1.47	16.517	0.351	20.123
SMHS	**60**	[1-2] case	22.47	1.72	103.102	1.885	530.642
SMHS	**61**	[1-2] case	11.32	0.90	57.257	1.311	341.770
SMHS	**62**	[1-2] case	26.65	0.31	1.689	0.137	5.716
SMHS	**63**	[1-2] case	10.80	1.36	58.992	1.869	107.716
SMHS	**64**	[1-2] case	37.72	0.93	12.449	0.589	13.994
SMHS	**65**	[1-2] case	12.38	0.23	1.823	0.164	2.346
SMHS	**66**	[1-2] case	7.02	0.85	17.951	0.430	13.223
SMHS	**67**	[1-2] case	10.42	0.99	25.380	0.637	24.168
SMHS	**68**	[1-2] case	7.23	1.44	14.667	1.166	62.355
SMHS	**69**	[1-2] case	24.45	0.68	4.134	0.253	14.779
SWHS	**70**	[1-2] case	8.10	1.67	48.470	0.730	26.699
SWHS	**71**	[1-2] case	9.59	1.33	23.278	1.899	116.177
SWHS	**72**	[1-2] case	8.18	0.93	5.986	0.440	6.877
SCCS	**73**	[1-2] case	9.78	0.74	56.187	0.815	300.845
SCCS	**74**	[1-2] case	49.33	0.45	13.468	1.302	12.825
SWHS	**75**	[1-2] ctrl	7.05	0.31	1.286	0.065	10.795
SWHS	**76**	[1-2] ctrl	7.55	0.40	0.000	0.024	2.339
SWHS	**77**	[1-2] ctrl	22.07	0.34	1.725	0.332	57.805
SWHS	**78**	[1-2] ctrl	8.59	0.45	3.492	0.260	13.010
SWHS	**79**	[1-2] ctrl	11.96	0.99	29.546	2.091	190.436
SWHS	**80**	[1-2] ctrl	10.04	0.76	67.843	2.104	244.181
SMHS	**81**	[1-2] ctrl	25.22	2.43	3.107	0.858	15.778
SMHS	**82**	[1-2] ctrl	9.80	0.74	13.233	0.919	62.003
SMHS	**83**	[1-2] ctrl	17.96	0.62	5.741	1.323	12.072
SMHS	**84**	[1-2] ctrl	18.66	1.07	10.013	0.780	27.048
SMHS	**85**	[1-2] ctrl	10.02	0.68	73.249	1.673	286.395
SMHS	**86**	[1-2] ctrl	22.03	1.75	78.541	1.365	235.152
SMHS	**87**	[1-2] ctrl	24.07	0.45	3.954	0.059	10.761
SMHS	**88**	[1-2] ctrl	9.23	1.61	4.868	0.346	18.037
SMHS	**89**	[1-2] ctrl	10.53	0.40	3.496	1.033	50.902
SMHS	**90**	[1-2] ctrl	15.61	0.96	24.349	0.862	64.538
SMHS	**91**	[1-2] ctrl	23.94	0.74	3.469	0.137	12.049
SMHS	**92**	[1-2] ctrl	30.86	0.42	2.917	0.492	9.805
SMHS	**93**	[1-2] ctrl	19.08	0.17	6.513	0.043	7.895
SMHS	**94**	[1-2] ctrl	9.49	0.17	3.050	0.216	20.764
SMHS	**95**	[1-2] ctrl	9.49	0.48	30.760	0.361	12.845
SMHS	**96**	[1-2] ctrl	14.26	1.64	98.251	2.337	106.489
SMHS	**97**	[1-2] ctrl	46.78	0.51	48.144	1.017	58.010
SMHS	**98**	[1-2] ctrl	7.45	1.78	41.460	0.551	4.203
SMHS	**99**	[1-2] ctrl	7.65	0.88	27.467	0.668	41.242
SMHS	**100**	[1-2] ctrl	69.37	0.76	40.096	0.417	16.711
SMHS	**101**	[1-2] ctrl	12.59	0.68	15.589	1.123	24.600
SMHS	**102**	[1-2] ctrl	14.31	0.57	5.266	0.489	7.312
SMHS	**103**	[1-2] ctrl	8.78	0.85	12.930	0.605	49.657
SMHS	**104**	[1-2] ctrl	7.76	0.82	34.398	0.911	95.563
SWHS	**105**	[1-2] ctrl	312.69	1.16	42.166	0.557	162.818
SWHS	**106**	[1-2] ctrl	11.62	1.07	61.745	0.588	149.490
SWHS	**107**	[1-2] ctrl	11.03	0.45	15.234	0.357	133.308
SWHS	**108**	[1-2] ctrl	8.10	1.19	34.752	1.244	79.109
SWHS	**109**	[1-2] ctrl	12.40	0.85	5.123	0.453	6.789

continue

SWHS	**110**	[1-2] ctrl	26.25	0.42	20.696	0.643	111.460
SCCS	**111**	[1-2] ctrl	16.69	0.71	6.821	0.602	24.728
SCCS	**112**	[1-2] ctrl	18.07	1.19	43.820	2.409	34.322
SCCS	**113**	[1-2] ctrl	9.24	1.78	14.689	1.188	35.882
SCCS	**114**	[1-2] ctrl	23.60	1.33	56.756	0.860	19.063
SWHS	**115**	[2-3] case	8.29	1.33	24.784	0.230	66.261
SWHS	**116**	[2-3] case	6.82	0.20	0.211	0.120	6.366
SWHS	**117**	[2-3] case	11.84	0.51	0.508	0.055	8.379
SWHS	**118**	[2-3] case	22.07	0.62	46.857	0.782	131.290
SWHS	**119**	[2-3] case	13.38	0.68	9.027	0.603	37.500
SMHS	**120**	[2-3] case	12.60	1.50	64.657	1.328	75.000
SMHS	**121**	[2-3] case	8.13	0.17	0.380	0.025	5.398
SMHS	**122**	[2-3] case	33.64	1.58	55.810	1.918	103.142
SMHS	**123**	[2-3] case	10.39	0.17	0.327	0.033	5.644
SMHS	**124**	[2-3] case	19.48	1.53	18.332	0.443	16.489
SMHS	**125**	[2-3] case	29.85	0.25	0.495	0.105	0.000
SMHS	**126**	[2-3] case	20.43	1.64	49.605	0.759	116.681
SMHS	**127**	[2-3] case	13.94	2.06	44.108	0.948	56.173
SMHS	**128**	[2-3] case	54.26	1.33	57.040	1.129	125.881
SMHS	**129**	[2-3] case	9.12	0.82	21.231	1.099	38.724
SCCS	**130**	[2-3] case	31.32	0.54	8.206	0.364	33.018
SCCS	**131**	[2-3] case	14.06	1.81	12.661	0.175	6.898
SCCS	**132**	[2-3] case	15.32	0.82	26.469	1.740	19.063
SCCS	**133**	[2-3] case	31.06	0.88	23.110	1.470	11.882
SCCS	**134**	[2-3] case	17.25	0.17	3.204	0.432	63.073
SCCS	**135**	[2-3] case	15.04	0.17	3.297	0.145	3.946
SWHS	**136**	[2-3] ctrl	7.12	0.42	0.487	0.165	1.534
SWHS	**137**	[2-3] ctrl	8.84	0.28	10.093	0.516	106.426
SWHS	**138**	[2-3] ctrl	39.28	1.56	5.357	0.095	2.239
SWHS	**139**	[2-3] ctrl	7.92	1.72	30.985	0.245	106.225
SWHS	**140**	[2-3] ctrl	10.04	0.68	18.854	1.767	98.775
SWHS	**141**	[2-3] ctrl	10.16	0.85	10.913	0.404	37.672
SWHS	**142**	[2-3] ctrl	22.35	0.28	5.320	0.084	50.960
SWHS	**143**	[2-3] ctrl	9.30	0.65	18.501	0.862	156.053
SWHS	**144**	[2-3] ctrl	15.86	0.68	10.550	0.255	43.059
SWHS	**145**	[2-3] ctrl	5.96	0.62	44.600	0.951	186.184
SMHS	**146**	[2-3] ctrl	7.79	0.51	30.444	0.462	13.458
SMHS	**147**	[2-3] ctrl	15.74	0.88	21.099	0.593	42.160
SMHS	**148**	[2-3] ctrl	13.96	1.07	34.324	1.432	26.134
SMHS	**149**	[2-3] ctrl	8.13	0.62	10.124	2.105	233.201
SMHS	**150**	[2-3] ctrl	16.94	1.89	57.050	2.134	118.915
SMHS	**151**	[2-3] ctrl	15.74	0.31	4.009	0.098	9.516
SMHS	**152**	[2-3] ctrl	15.48	0.23	6.569	0.461	14.868
SMHS	**153**	[2-3] ctrl	22.63	0.45	55.780	0.368	119.604
SMHS	**154**	[2-3] ctrl	8.84	1.81	61.433	0.092	85.707
SMHS	**155**	[2-3] ctrl	46.48	0.17	3.493	0.108	8.295
SMHS	**156**	[2-3] ctrl	11.58	1.67	11.451	1.463	48.094
SMHS	**157**	[2-3] ctrl	28.84	1.07	29.578	1.461	6.564
SMHS	**158**	[2-3] ctrl	11.50	0.37	0.942	0.096	0.384
SMHS	**159**	[2-3] ctrl	9.83	1.87	14.156	1.252	18.389
SMHS	**160**	[2-3] ctrl	8.66	1.36	27.961	0.502	100.740
SMHS	**161**	[2-3] ctrl	33.80	0.88	22.395	0.360	16.124
SMHS	**162**	[2-3] ctrl	10.30	1.64	38.834	0.871	84.571
SMHS	**163**	[2-3] ctrl	9.47	1.33	18.410	0.535	153.989
SMHS	**164**	[2-3] ctrl	14.43	0.37	17.380	0.445	81.437
SMHS	**165**	[2-3] ctrl	43.19	0.20	2.809	0.148	21.844
SCCS	**166**	[2-3] ctrl	12.40	0.62	5.410	0.588	13.383

continue

SCCS	**167**	[2-3] ctrl	11.01	0.59	2.089	0.290	9.095
SCCS	**168**	[2-3] ctrl	26.99	1.10	57.229	0.563	27.811
SCCS	**169**	[2-3] ctrl	12.35	1.56	18.299	1.706	50.634
SCCS	**170**	[2-3] ctrl	31.32	1.13	2.492	0.546	4.844
SCCS	**171**	[2-3] ctrl	13.23	0.93	28.508	1.311	36.086
SCCS	**172**	[2-3] ctrl	15.86	0.68	21.161	1.203	7.947
SCCS	**173**	[2-3] ctrl	103.86	0.42	19.036	0.542	19.168
SCCS	**174**	[2-3] ctrl	47.14	1.22	58.924	3.318	65.760
SCCS	**175**	[2-3] ctrl	14.89	1.19	43.479	4.399	745.393
SCCS	**176**	[2-3] ctrl	9.38	1.95	19.991	2.613	53.957
SCCS	**177**	[2-3] ctrl	15.86	1.72	11.286	2.257	20.853
SWHS	**178**	[3-4] case	11.06	0.82	25.543	0.667	53.275
SWHS	**179**	[3-4] case	11.57	0.34	4.879	0.572	7.236
SMHS	**180**	[3-4] case	21.78	0.17	0.236	0.073	1.854
SMHS	**181**	[3-4] case	39.45	0.62	3.859	0.256	35.973
SMHS	**182**	[3-4] case	23.55	0.96	23.476	1.254	26.446
SMHS	**183**	[3-4] case	24.07	1.41	12.708	0.260	7.609
SMHS	**184**	[3-4] case	7.97	0.71	4.156	0.083	2.048
SMHS	**185**	[3-4] case	10.13	0.76	10.709	0.409	17.735
SMHS	**186**	[3-4] case	23.03	0.62	10.276	0.233	15.111
SMHS	**187**	[3-4] case	13.73	1.53	25.765	0.442	93.744
SMHS	**188**	[3-4] case	10.26	1.05	76.100	0.801	112.658
SMHS	**189**	[3-4] case	15.20	1.24	41.175	0.285	20.926
SMHS	**190**	[3-4] case	21.42	1.67	33.058	1.137	33.967
SMHS	**191**	[3-4] case	27.59	0.23	10.240	0.342	3.141
SMHS	**192**	[3-4] case	16.76	1.44	38.487	1.700	14.691
SMHS	**193**	[3-4] case	6.72	0.79	11.702	1.361	53.516
SMHS	**194**	[3-4] case	9.01	0.17	0.106	0.004	1.118
SWHS	**195**	[3-4] case	8.21	1.16	26.549	0.867	25.957
SWHS	**196**	[3-4] case	8.44	0.62	9.660	0.650	94.906
SCCS	**197**	[3-4] case	8.58	1.61	5.820	0.665	169.583
SCCS	**198**	[3-4] case	17.86	0.54	38.136	2.498	68.124
SCCS	**199**	[3-4] case	16.42	0.25	18.283	1.359	124.394
SCCS	**200**	[3-4] case	9.38	0.54	34.581	3.536	145.117
SWHS	**201**	[3-4] ctrl	9.68	0.90	32.872	0.193	128.572
SWHS	**202**	[3-4] ctrl	15.05	0.51	2.558	0.062	31.330
SWHS	**203**	[3-4] ctrl	35.42	0.28	18.164	0.239	6.165
SWHS	**204**	[3-4] ctrl	11.44	1.33	16.795	0.308	23.547
SMHS	**205**	[3-4] ctrl	9.80	1.87	50.505	1.259	110.550
SMHS	**206**	[3-4] ctrl	11.57	1.89	53.462	1.169	184.875
SMHS	**207**	[3-4] ctrl	7.73	0.48	0.705	0.731	1.587
SMHS	**208**	[3-4] ctrl	22.76	1.22	16.718	1.515	39.382
SMHS	**209**	[3-4] ctrl	14.66	0.23	7.711	0.110	78.364
SMHS	**210**	[3-4] ctrl	6.01	1.22	39.391	2.508	57.985
SMHS	**211**	[3-4] ctrl	12.92	0.59	12.468	0.257	12.496
SMHS	**212**	[3-4] ctrl	10.66	1.87	17.022	0.434	53.095
SMHS	**213**	[3-4] ctrl	10.01	1.24	33.057	0.694	136.012
SMHS	**214**	[3-4] ctrl	10.13	0.42	1.864	0.078	7.609
SMHS	**215**	[3-4] ctrl	11.05	1.27	47.877	0.203	95.811
SMHS	**216**	[3-4] ctrl	9.49	0.37	26.343	0.237	36.251
SMHS	**217**	[3-4] ctrl	10.92	2.09	53.751	0.785	70.925
SMHS	**218**	[3-4] ctrl	9.49	0.82	26.874	0.632	93.516
SMHS	**219**	[3-4] ctrl	12.25	1.13	28.006	0.167	69.148
SMHS	**220**	[3-4] ctrl	6.01	0.90	10.241	0.327	22.223
SMHS	**221**	[3-4] ctrl	13.86	0.85	26.396	0.616	38.840
SMHS	**222**	[3-4] ctrl	9.61	0.90	27.680	0.604	72.221
SMHS	**223**	[3-4] ctrl	8.22	0.76	17.298	1.175	24.282

continue

SMHS	**224**	[3-4] ctrl	69.57	0.68	6.987	0.033	0.384
SMHS	**225**	[3-4] ctrl	31.77	1.10	14.886	1.085	3.596
SMHS	**226**	[3-4] ctrl	7.76	2.32	65.897	1.864	39.825
SMHS	**227**	[3-4] ctrl	37.34	1.58	92.279	2.659	382.510
SMHS	**228**	[3-4] ctrl	22.02	1.10	38.041	0.706	119.292
SMHS	**229**	[3-4] ctrl	8.21	1.61	42.351	3.082	134.718
SMHS	**230**	[3-4] ctrl	7.02	0.54	4.769	0.883	29.363
SMHS	**231**	[3-4] ctrl	8.32	1.92	55.830	1.736	231.876
SMHS	**232**	[3-4] ctrl	11.98	1.39	65.045	1.600	318.197
SMHS	**233**	[3-4] ctrl	12.47	0.17	1.289	0.131	4.917
SMHS	**234**	[3-4] ctrl	16.52	0.76	33.155	1.234	71.378
SWHS	**235**	[3-4] ctrl	9.01	1.24	12.515	0.528	111.460
SWHS	**236**	[3-4] ctrl	7.76	1.58	37.355	1.525	112.045
SWHS	**237**	[3-4] ctrl	11.43	1.44	18.092	0.656	32.072
SWHS	**238**	[3-4] ctrl	7.41	0.65	15.310	0.188	79.987
SCCS	**239**	[3-4] ctrl	9.24	0.54	10.418	0.617	16.985
SCCS	**240**	[3-4] ctrl	29.13	1.87	67.431	2.831	290.621
SCCS	**241**	[3-4] ctrl	8.55	2.06	5.232	0.467	9.226
SCCS	**242**	[3-4] ctrl	7.55	0.54	11.009	0.454	6.116
SCCS	**243**	[3-4] ctrl	20.37	1.78	117.313	3.205	63.348
SCCS	**244**	[3-4] ctrl	9.11	2.88	100.121	3.695	164.868
SCCS	**245**	[3-4] ctrl	31.95	0.17	2.283	0.450	21.811
SCCS	**246**	[3-4] ctrl	46.90	0.68	34.744	2.000	15.219
SWHS	**247**	[4-5] case	17.26	0.51	9.276	0.065	47.638
SWHS	**248**	[4-5] case	6.95	1.07	20.537	0.213	148.604
SWHS	**249**	[4-5] case	17.68	0.54	15.648	0.173	18.063
SMHS	**250**	[4-5] case	29.09	0.59	56.340	2.504	737.340
SMHS	**251**	[4-5] case	8.98	0.51	55.311	2.308	298.871
SMHS	**252**	[4-5] case	11.32	1.36	81.368	1.936	703.571
SMHS	**253**	[4-5] case	18.28	0.82	7.847	1.004	17.004
SMHS	**254**	[4-5] case	18.54	0.96	75.342	1.017	52.160
SMHS	**255**	[4-5] case	7.34	1.75	40.531	1.362	84.891
SMHS	**256**	[4-5] case	9.70	0.96	14.742	0.397	36.766
SWHS	**257**	[4-5] case	11.62	1.44	19.414	0.876	59.352
SCCS	**258**	[4-5] case	10.60	0.79	32.140	0.448	67.743
SCCS	**259**	[4-5] case	26.37	0.23	1.854	0.163	9.791
SCCS	**260**	[4-5] case	13.64	0.62	17.929	0.458	35.707
SCCS	**261**	[4-5] case	7.29	0.42	9.949	0.891	34.485
SCCS	**262**	[4-5] case	9.38	0.48	4.235	0.348	18.589
SCCS	**263**	[4-5] case	12.35	0.76	0.847	0.454	3.861
SWHS	**264**	[4-5] ctrl	10.80	1.56	77.719	1.086	161.388
SWHS	**265**	[4-5] ctrl	12.36	0.45	0.593	0.162	1.232
SWHS	**266**	[4-5] ctrl	26.68	0.82	37.039	0.230	119.714
SWHS	**267**	[4-5] ctrl	8.59	1.05	15.672	0.675	74.515
SWHS	**268**	[4-5] ctrl	13.83	0.54	28.715	0.556	29.720
SWHS	**269**	[4-5] ctrl	22.35	0.40	2.927	1.230	9.542
SMHS	**270**	[4-5] ctrl	11.18	0.51	2.830	0.159	3.297
SMHS	**271**	[4-5] ctrl	31.49	1.13	103.841	1.544	645.582
SMHS	**272**	[4-5] ctrl	9.10	1.16	45.385	2.657	147.280
SMHS	**273**	[4-5] ctrl	8.09	1.44	34.200	2.320	45.151
SMHS	**274**	[4-5] ctrl	12.38	1.05	14.145	0.421	21.033
SMHS	**275**	[4-5] ctrl	18.68	0.31	2.264	0.164	12.049
SMHS	**276**	[4-5] ctrl	9.49	1.67	18.819	0.857	148.730
SMHS	**277**	[4-5] ctrl	8.47	0.62	7.943	1.418	23.845
SMHS	**278**	[4-5] ctrl	18.54	0.76	19.627	1.871	31.184
SMHS	**279**	[4-5] ctrl	6.39	0.48	8.460	0.714	17.630
SMHS	**280**	[4-5] ctrl	7.23	1.02	19.873	2.165	13.867

continue

SMHS	**281**	[4-5] ctrl	9.83	0.57	33.174	1.227	110.398
SMHS	**282**	[4-5] ctrl	29.27	0.90	68.150	0.208	86.667
SMHS	**283**	[4-5] ctrl	19.83	0.34	5.655	0.240	35.955
SWHS	**284**	[4-5] ctrl	22.75	0.65	6.666	0.101	12.855
SWHS	**285**	[4-5] ctrl	10.66	1.33	45.285	1.402	89.007
SCCS	**286**	[4-5] ctrl	11.57	0.79	9.519	0.418	27.324
SCCS	**287**	[4-5] ctrl	12.40	0.31	32.823	1.012	76.841
SCCS	**288**	[4-5] ctrl	5.96	0.96	43.679	1.456	57.845
SCCS	**289**	[4-5] ctrl	18.07	0.31	3.426	0.105	19.072
SCCS	**290**	[4-5] ctrl	10.88	0.51	10.892	0.874	41.169
SCCS	**291**	[4-5] ctrl	12.67	0.17	0.113	0.089	12.178
SCCS	**292**	[4-5] ctrl	14.48	0.82	58.180	1.182	38.070
SCCS	**293**	[4-5] ctrl	14.48	0.45	8.888	0.875	26.268
SCCS	**294**	[4-5] ctrl	11.29	0.28	0.129	0.345	1.558
SCCS	**295**	[4-5] ctrl	13.23	1.22	15.833	0.832	86.133
SCCS	**296**	[4-5] ctrl	6.53	0.42	15.051	1.903	28.279
SCCS	**297**	[4-5] ctrl	7.12	1.22	52.368	3.708	110.605

Supporting Information Table S2. Statistical differences in controls between SCCS and SM/WHS cohorts.

	SCCS control (n=44)	SMHS control (n=104)	SWHS control (n=50)	*P*
**Age, Median (IQR)** (years)	57 (51-62)	66.97 (57.23-71.91)	64.91 (58.19-67.31)	*<0.001*
**CA19-9, Median (IQR)**	14.35 (9.68-28.33)	10.36 (8.53-15.49)	11.23 (9.32-15.73)	*0.018*
**Creatinine, Median (IQR)**	1.16 (0.56-1.67)	0.82 (0.47-1.19)	0.66 (0.42-1.03)	*0.017*
**LYVE1 R&D, Median (IQR)**	18.13 (9.56-41.97)	18.17 (6.64-34.34)	10.32 (3.68-29.34)	*0.081*
**TFF1 R&D, Median (IQR)**	1.11 (0.45-1.93)	0.63 (0.35-1.02)	0.39 (0.2-0.89)	*0.008*
**REG1B Sino, Median (IQR)**	20.89 (7.85-51.46)	46.26 (16.04-95.07)	53.83 (13.56-104.36)	*0.035*
**Diabetes at Enrolment**				
Diabetes ≤3 years	4 (9.1%)	3 (2.9%)	0 (0%)	
Diabetes >3 years	10 (22.7%)	4 (3.8%)	4 (8%)	*<0.001*
No diabetes	30 (68.2%)	97 (93.3%)	46 (92%)	
**Familial cancer**				
1st degree	23 (52.3%)	30 (28.8%)	14 (28%)	*0.017*
No FC	21 (47.7%)	74 (71.2%)	36 (72%)	
**Smoking**				
Current	17 (39.5%)	47 (45.2%)	2 (4%)	
Former	15 (34.9%)	16 (15.4%)	1 (2%)	*<0.001*
Never	11 (25.6%)	41 (39.4%)	47 (94%)	
Unknown	*0*	*0*	*1*	
**Drinking**				
Heavy	6 (15.4%)	5 (4.9%)	0 (0%)	
Moderate	16 (41%)	30 (29.1%)	1 (2%)	*<0.001*
Never	17 (43.6%)	68 (66%)	48 (98%)	
Unknown	*5*	*1*	*1*	
**BMI Enrollment, Median (IQR)**	29.7 (26.2-34.1)	23.8 (21.4-25.9)	24.2 (21.9-26.8)	*<0.001*
**BMI Peak, Median (IQR)**	31.8 (27.9-37.5)	23.8 (21.8-25.9)	24.4 (22.2-27.8)	*<0.001*
**Hypertension**				
Yes	27 (61.4%)	36 (34.6%)	22 (44%)	*0.011*
No	17 (38.6%)	68 (65.4%)	28 (56%)	
**Asthma**				
No	36 (81.8%)	102 (98.1%)	50 (100%)	*<0.001*
Yes	8 (18.2%)	2 (1.9%)	0 (0%)	
**Education**				
Less than High School	13 (29.5%)	54 (51.9%)	34 (68%)	
High school	17 (38.6%)	32 (30.8%)	9 (18%)	*0.005*
Vocational training/ some college	9 (20.5%)	6 (5.8%)	4 (8%)	
College graduate	5 (11.4%)	12 (11.5%)	3 (6%)	

IQR, Interquartile Range; FC, familial cancer

Supporting Information Table S3. Distribution of possible confounders by four groups for each urinary biomarker. Group 1: Controls with biomarker ≥ upper quartiles of controls; Group 2: Cases with biomarker ≥ upper quartiles of controls; Group 3: Controls with biomarker < upper quartiles of controls; Group 4: Cases with biomarker < upper quartiles of controls.

(A) LYVE1

**Groups**		**Group 1 n (%)**	**Group 2 n (%)**	**Group 3 n (%)**	**Group 4 n (%)**	***P***
N in each group		38 (ctrls)	28 (cases)	116 (ctrls)	49 (cases)	
Fast sample	≥8 h	3 (7.9)	7 (25.0)	19 (16.4)	5 (10.2)	*0.18*
Comorbidity [a]	≥1	12 (31.6)	10 (35.7)	36 (31.0)	18 (36.7)	*0.89*
Comorbidity [a]	0	26 (68.4)	18 (64.3)	80 (69.0)	31 (63.3)	
	1	5 (13.2)	3 (10.7)	19 (16.4)	7 (14.3)	
	2	5 (13.2)	3 (10.7)	12 (10.3)	4 (8.2)	
	3	1 (2.6)	2 (7.1)	3 (2.6)	4 (8.2)	
	4	1 (2.6)	1 (3.6)	1 (0.9)	1 (2.0)	
	≥5	0	1 (3.6)	1 (0.9)	2 (4.1)	
Sample collection	afternoon	22 (57.9)	14 (50.0)	66 (56.9)	30 (61.2)	*0.81*
NSAID used [b]	yes	3 (7.9)	2 (7.1)	21 (18.1)	5 (10.2)	
Antibiotics [b]	yes	0 (0.0)	1 (3.6)	10 (8.6)	4 (8.1)	
Hormone [b]	yes	2 (5.3)	3 (10.7)	2 (1.7)	0 (0.0)	
Anti-hypertension [b]	yes	6 (15.8)	4 (14.3)	20 (17.2)	10 (20.4)	
Gender	male	25 (65.8)	23(82.1)	79 (68.1)	29 (59.2)	*0.23*

(B) TFF1

**Groups**		**Group 1 n (%)**	**Group 2 n (%)**	**Group 3 n (%)**	**Group 4 n (%)**	***P***
N in each group		38	22	116	55	
Fast sample	≥8 h	8 (21.0)	5 (22.7)	14 (12.1)	7 (12.7)	*0.37*
Comorbidity [a]	≥1	12 (31.6)	7 (31.8)	36 (31.0)	21 (38.2)	*0.82*
Comorbidity [a]	0	26 (68.4)	15 (68.2)	80 (69.0)	34 (61.8)	
	1	5 (13.2)	3 (13.6)	19 (16.4)	7 (12.7)	
	2	6 (15.8)	1 (4.5)	11 (9.5)	6 (10.9)	
	3	1 (2.6)	1 (4.5)	3 (2.6)	5 (9.1)	
	4	0	1 (4.5)	2 (1.7)	1 (1.8)	
	≥5	0	1 (4.5)	1 (0.9)	2 (3.6)	
Sample collection	afternoon	21 (55.3)	13 (59.1)	67 (57.8)	31 (56.4)	*0.99*
NSAID used [b]	yes	4 (10.5)	1 (4.5)	20 (17.2)	6 (10.9)	
Antibiotics [b]	yes	0	1 (4.5)	10 (8.6)	4 (7.3)	
Hormone [b]	yes	1 (2.6)	2 (9.1)	3 (2.6)	1 (1.8)	
Anti-hypertension [b]	yes	5 (13.2)	2 (9.1)	21 (18.1)	12 (21.8)	
Gender	male	25 (65.8)	15 (68.2)	79 (68.1)	37 (67.3)	*0.99*

(C) REG1B

**Groups**		**Group 1 n (%)**	**Group 2 n (%)**	**Group 3 n (%)**	**Group 4 n (%)**	***P***
N in each group		45	25	109	52	
Fast sample	≥8 h	7 (15.6)	7 (28.0)	15 (13.8)	5 (9.6)	*0.20*
Comorbidity [a]	≥1	15 (33.3)	10 (40.0)	33 (30.3)	18 (34.6)	*0.81*
Comorbidity [a]	0	30 (66.7)	15 (60.0)	76 (69.7)	34 (65.4)	
	1	8 (17.8)	4 (16.0)	16 (14.7)	6 (11.5)	
	2	6 (13.3)	1 (4.0)	11 (10.1)	6 (11.5)	
	3	0	3 (12.0)	4 (3.7)	3 (5.8)	
	4	1 (2.2)	1 (4.0)	1 (0.9)	1 (1.9)	
	≥5	0	1 (4.0)	1 (0.9)	2 (3.8)	
Sample collection	afternoon	23 (51.1)	13 (52.0)	65 (59.6)	31 (59.6)	*0.72*
NSAID used [b]	yes	3 (6.7)	1 (4.0)	21 (19.3)	6 (11.5)	
Antibiotics [b]	yes	1 (2.2)	0	9 (8.3)	5 (9.6)	
Hormone [b]	yes	2 (4.4)	1 (4.0)	2 (1.8)	2 (3.8)	
Anti-hypertension [b]	yes	5 (11.1)	3 (12.0)	21 (19.3)	11 (21.1)	
Gender	male	29 (64.4)	16 (64.0)	75 (68.8)	36 (69.2)	*0.92*

[a] Comorbidities include coronary heart disease (CHD) or acute myocardial infarction (AMI), diabetes, cancer, stroke, hepatitis, chronic pulmonary disease and gastrointestinal ulcer disease.

[b] Used within a week before sample collection.

Supporting Information Table S4. Covariate analysis.

(A) Shanghai cohorts

	LYVE1 median (IQR)			REG1B median (IQR)			TFF1 median (IQR)		
	**Cases**	**Controls**	***P***	**Cases**	**Controls**	***P***	**Cases**	**Controls**	***P***
**Diabetes**									
Yes	30.1 (14.7-51.3)	19 (7.8-44.2)	*0.536*	79.2 (38.7-132.6)	90 (51.8-160)	*0.979*	0.72 (0.37-1.21)	0.67 (0.48-1)	*0.936*
No	19.8 (12.3-43)	19.5 (8.8-33.2)	*0.468*	65 (28.2-133.7)	51.7 (25-104.4)	*0.274*	0.89 (0.42-1.47)	0.73 (0.41-1.33)	*0.468*
**Familial cancer**									
1st degree	27.4 (16.7-53.6)	21.3 (8.5-33)	*0.046*	79.9 (38.4-174.6)	54.2 (31.4-106)	*0.241*	0.77 (0.37-2)	0.64 (0.45-1.06)	*0.575*
No FC	18.2 (10.8-36.6)	19.4 (8.9-33.4)	*0.974*	61.3 (28.2-118.8)	55.9 (24-114.3)	*0.456*	0.89 (0.45-1.4)	0.77 (0.37-1.48)	*0.913*
**Smoking**									
Current/ Former	19.8 (8.3-43.2)	23.4 (10.2-34.4)	*0.736*	43.1 (17.7-117)	58.8 (29.6-114)	*0.319*	0.81 (0.44-1.39)	0.72 (0.44-1.14)	*0.62*
Never	20.6 (13.8-44.8)	17.8 (7.2-31.4)	*0.076*	70.6 (54.3-138.9)	47.4 (23.9-105)	***0.015***	0.81 (0.34-1.43)	0.72 (0.36-1.4)	*0.785*
**Drinking in men**									
Heavy/ Moderate	36.4 (10.5-54.4)	25.7 (10.2-42.6)	*0.693*	39.4 (21-177.9)	61.2 (39-127.1)	*0.461*	1.05 (0.44-2.07)	0.74 (0.37-1.52)	*0.309*
Never	23.3 (12.2-46)	21.3 (9.9-30.8)	*0.237*	63.4 (28.6-106.9)	46.1 (22.2-91.9)	*0.052*	0.77 (0.46-1.29)	0.74 (0.44-1.18)	*0.705*
**Drinking in women**									
Heavy/ Moderate	73 Never, 1 Moderate, 1 Unknown								
Never									
**Hypertension**									
Yes	17.5 (6.9-36.6)	21.6 (8.7-44)	*0.534*	58.6 (21.9-94.1)	62.4 (23.5-137)	*0.723*	0.63 (0.23-1.25)	0.73 (0.4-1.68)	*0.174*
No	23.6 (14.1-44.8)	19.4 (9.1-30.9)	*0.046*	70.9 (33.5-142)	50 (28.9-99.8)	*0.065*	0.97 (0.53-1.51)	0.72 (0.41-1.18)	*0.075*
**Asthma**									
Yes	17.1 (15.5-17.9)	38.3 (35.2-41.3)	*0.2*	128.5 (89.1-135.2)	133.2 (78.8-188)	*1*	0.17 (0.15-0.18)	0.85 (0.61-1.1)	*0.2*
No	22.1 (12.2-43.6)	19.4 (8.8-33)	*0.2*	65 (29.6-131.5)	54.7 (25.1-109)	*0.272*	0.87 (0.45-1.45)	0.72 (0.41-1.29)	*0.313*

IQR, Inter Quartile Range; FC, familial cancer.

(B) SCCS cohort

	LYVE1 median (IQR)			REG1B median (IQR)			TFF1 median (IQR)		
	**Cases**	**Controls**	***P***	**Cases**	**Controls**	***P***	**Cases**	**Controls**	***P***
**Diabetes**									
Yes	27.6 (26.3-33.9)	23 (13.5-37.3)	*0.688*	13.7 (6.1-47.4)	19.6 (4.2-56.4)	*0.975*	0.6 (0.46-1.62)	1.02 (0.35-1.66)	*0.781*
No	29.9 (18.8-64)	15.7 (7.7-36.4)	*0.117*	26.8 (19.2-126.2)	22.8 (11-48)	*0.339*	1.23 (0.75-2.89)	0.79 (0.51-1.39)	*0.222*
**Familial cancer**									
1st degree	28.3 (15.9-65.7)	13.5 (6-30.9)	*0.141*	47.2 (17.7-161.8)	16.9 (4.4-37.2)	*0.062*	1.83 (1.14-3.1)	0.71 (0.47-1.37)	*0.013*
No FC	29.2 (26.8-33.2)	23.3 (11.7-42.7)	*0.884*	22 (8-36.9)	32 (12.1-67.3)	*0.268*	0.51 (0.17-0.79)	1.09 (0.65-1.55)	*0.065*
**Smoking**									
Current/ Former	29.2 (19-56.1)	20.5 (6.8-36.9)	*0.133*	25 (14.6-103.1)	25 (9.1-54.7)	*0.489*	1.3 (0.54-2.4)	1.1 (0.6-1.6)	*0.464*
Never	30.1 (20-35.1)	19.8 (11.8-39.3)	*1*	26.6 (11.4-41.6)	20.3 (10.9-55.3)	*0.95*	0.57 (0.42-0.87)	0.68 (0.47-1.36)	*0.753*
**Drinking**									
Heavy/ Moderate	30.2 (22.6-64.1)	15.7 (8.8-35.6)	*0.166*	66.1 (18.2-191.2)	30.4 (13.5-79.7)	*0.375*	1.23 0.67-2.3)	1.19 (0.72-1.51)	*0.836*
Never	29.4 (21.2-37)	20.5 (5.1-38.2)	*0.431*	23.2 (16.1-35.6)	15.6 (4.5-35.6)	*0.516*	0.7 (0.43-1.8)	0.51 (0.42-1.29)	*0.609*

**Hypertension**									
Yes	30.2 8.6-36.2)	21.1 10.9-42.8)	*0.856*	26.8 9.4-68.6)	20.3 (7.2-47.2)	*0.42*	0.6 (0.43-1.81)	0.71 (0.49-1.49)	*0.604*
No	28.4 (27-47)	12 (6.6-31.1)	*0.089*	23.2 15.3-148.6)	27.7 (10.8-83.9)	*0.664*	1.67 (1.11-4.2)	1.09 (0.48-1.41)	*0.099*
**Asthma**									
Yes	30.8 27.6-32.3)	10.9 5.7-13.5)	*0.002*	23.2 (13.7-39.7)	30.6 (4.2-84.7)	*0.943*	0.55 (0.48-1.37)	0.68 (0.43-1.15)	*0.724*
No	28.4 (10.3-64)	23 (8.2-42.7)	*0.657*	26.8 (13.5-113.7)	20.3 (10.6-50.1)	*0.369*	1.23 (0.6-2.5)	1.02 (0.51-1.58)	*0.503*

IQR, Inter Quartile Range; FC, familial cancer.

Supporting Information Table S5. Variable comparison by categories in pooled cases and controls.

Categories	Controls (n=198)	Cases 0-1 year (n=18)	*P*	Cases 0-2 years (n=38)	*P*	Cases 0-5 years (n=99)	*P*
**Diabetes at Enrolment**							
Diabetes	25 (12.63%)	4 (22.22%)	*0.434*	8 (21.05%)	*0.264*	23 (23.23%)	*0.03*
No diabetes	173 (87.37%)	14 (77.78%)		30 (78.95%)		76 (76.77%)	
**Familiar cancer**							
1st degree family	67 (33.84%)	6 (33.33%)	*1*	11(28.95%)	*0.69*	34 (34.34%)	*1*
No familiar cancer	131 (66.16%)	12 (66.67%)		27(71.05%)		65 (65.66%)	
**Smoking**							
Current	66 (33.5%)	7 (38.89%)	*0.812*	15(39.47%)	*0.604*	41 (41.41%)	*0.167*
Former	32 (16.24%)	2 (11.11%)		4(10.53%)		9 (9.09%)	
Never	99 (50.25%)	9 (50%)		19(50.00%)		49 (49.49%)	
Unknown	*1*						
**Drinking**							
Heavy	11 (5.76%)	3 (16.67%)	*0.185*	3(8.33%)	*0.193*	7 (7.45%)	*0.54*
Moderate	47 (24.61%)	3 (16.67%)		4(11.11%)		18 (19.15%)	
Never	133 (69.63%)	12 (66.67%)		29(80.56%)		69 (73.4%)	
Unknown	*7*			*2*		*5*	
**BMI at Enrolment**							
Median (IQR)	24.85 (22.18-27.73)	22.02 (19.27-25.68)	*0.027*	23.38 (21-25.9)	*0.04*	23.74 (21.58-26.88)	*0.112*
**Hypertension**							
Yes	85 (42.93%)	10 (55.56%)	*0.432*	15(39.47%)	*0.829*	43 (43.43%)	*1*
No	113 (57.07%)	8 (44.44%)		23(60.53%)		56 (56.57%)	
**Asthma**							
No	188 (94.95%)	16 (88.89%)	*0.591*	35(92.11%)	*0.752*	91 (91.92%)	*0.439*
Yes	10 (5.05%)	2 (11.11%)		3(7.89%)		8 (8.08%)	
**Education**							
Less than high school	101 (51.01%)	9 (50%)	*0.94*	21(55.26%)	*0.796*	46 (46.46%)	*0.427*
High school	58 (29.29%)	6 (33.33%)		9(23.68%)		28 (28.28%)	
Vocational training/ some college	19 (9.6%)	1 (5.56%)		5(13.16%)		16 (16.16%)	
College graduate	20 (10.1%)	2 (11.11%)		3(7.89%)		9 (9.09%)	

IQR, Interquartile Range
